# Prevalence and factors associated with mental health problems of psychological distress and depression among rural Victorians – analysis of cross-sectional data (Crossroads II)

**DOI:** 10.1186/s12888-023-04931-5

**Published:** 2023-06-20

**Authors:** Anushka Dashputre, Kingsley E. Agho, Milan K. Piya, Kristen Glenister, Lisa Bourke, Stephanie Hannah, Ravi Bhat, Uchechukwu L. Osuagwu, David Simmons

**Affiliations:** 1 School of Medicine, Western Sydney University, Campbelltown, NSW 2560 Australia; 2 School of Health Sciences, Western Sydney University, Campbelltown, NSW 2560 Australia; 3 Translational Health Research Institute (THRI), School of Medicine, Western Sydney University, Campbelltown, NSW 2560 Australia; 4 Macarthur Diabetes Endocrinology Metabolism Services, Camden and Campbelltown Hospitals, Campbelltown, NSW 2560 Australia; 5 Department of Rural Health, University of Melbourne, Wangaratta, VIC Australia; 6 Department of Rural Health, University of Melbourne, Shepparton, VIC Australia; 7 School of Science, Western Sydney University, Campbelltown, NSW 2560 Australia; 8 Bathurst Rural Clinical School (BRCS), School of Medicine, Western Sydney University, Bathurst, NSW 2795 Australia

**Keywords:** Mental health, Depression, Psychological distress, Lifestyle, Rural, Crossroads, Health, Regional

## Abstract

**Background:**

Research suggests that rates of mental illness are similar in rural and urban Australia, although there are significant workforce shortages in rural regions along with higher rates of chronic disease and obesity and lower levels of socioeconomic status. However, there are variations across rural Australia and limited local data on mental health prevalence, risk, service use and protective factors. This study describes the prevalence of self-reported mental health problems of psychological distress and depression, in a rural region in Australia and aims to identify the factors associated with these problems.

**Methods:**

The Crossroads II study was a large-scale cross-sectional study undertaken in the Goulburn Valley region of Victoria, Australia in 2016–18. Data were collected from randomly selected households across four rural and regional towns and then screening clinics from individuals from these households. The main outcome measures were self-reported mental health problems of psychological distress assessed by the Kessler 10 and depression assessed by Patient Health Questionnaire-9. Unadjusted odd ratios and 95% confidence intervals of factors associated with the two mental health problems were calculated using simple logistic regression with multiple logistic regression using hierarchical modelling to adjust for the potential confounders.

**Results:**

Of the 741 adult participants (55.6% females), 67.4% were aged ≥ 55 years. Based on the questionnaires, 16.2% and 13.6% had threshold-level psychological distress and depression, respectively. Of those with threshold-level K-10 scores, 19.0% and 10.5% had seen a psychologist or a psychiatrist respectively while 24.2% and 9.5% of those experiencing depression had seen a psychologist or a psychiatrist, respectively in the past year. Factors such as being unmarried, current smoker, obesity, were significantly associated with a higher prevalence of mental health problems whereas physical activity, and community participation reduced the risk of mental health problems. Compared to rural towns, the regional town had higher risk of depression which was non-significant after adjusting for community participation and health conditions.

**Conclusions:**

The high prevalence of psychological distress and depression in this rural population was consistent with other rural studies. Personal and lifestyle factors were more relevant to mental health problems than degree of rurality in Victoria. Targeted lifestyle interventions could assist in reducing mental illness risk and preventing further distress.

**Supplementary Information:**

The online version contains supplementary material available at 10.1186/s12888-023-04931-5.

## Introduction

Rural and regional towns are known to have demographic, social, economic, and environmental features distinct from capital cities that can uniquely influence the mental health and wellbeing of residents [1–3]. These include an older population, higher rates of chronic disease, lower socioeconomic status and reduced access to health and social services. For several decades, rural areas have also had severe workforce shortages, particularly in mental health, limiting access to care. These regions are also more prone to natural disasters including drought, flood and fire.

In conjunction with particular rural circumstances, the prevalence of mental and behavioural conditions has been steadily increasing nationwide, rising from 17.5% in 2014–15 to 20.1% in 2017–18 [4]. In 2018, mental and substance use disorders also caused the largest non-fatal burden for Australians aged under 50 [5]. This is of concern as psychological disorders have a high economic burden, with an estimated $11 billion spent nationally on mental health care in 2019–20 [6]. Mental health disorders also have known associations with chronic disease such as diabetes and respiratory disease [7, 8], adding to the overall burden of disease and impacting on quality of life.

Past studies examining urban/rural differences in the prevalence of mental health disorders have been cross-sectional in nature and have identified factors including age, gender and access to services that influence the prevalence of mental health disorders. A summary of the published literature [9–19] presented in the supplementary Table (S1) shows that under-representation of rural and regional participants is a key limitation of previous population surveys on mental health in Australia [10, 13]. Overall, the studies found that the prevalence of mental health conditions is similar between rural and urban residents in Australia, but access to mental health services is more limited in rural areas. This is supported by national health surveys showing that the prevalence of mental illness is around 20% and similar around the nation [20]. However, there is concern that a similar rate of mental illness between urban and rural populations masks a higher prevalence of psychological distress and undiagnosed or untreated mental illness in rural areas [21, 22] with one study suggesting that the prevalence of psychological distress in rural Australians may be as high as 31% [9]. With approximately 29% [23] of Australia’s population residing outside of capital cities, the health and wellbeing of these residents is of national importance. In addition, the use of limited sample sizes in the previous studies have led to the aggregation of rural, and regional towns, which does not accurately reflect the large variations in factors such as income, lifestyle, health outcomes, and healthcare accessibility that exist between small, rural towns and regional centres [10, 13]. These studies demonstrate the need for further investigation to distinguish any potential differences between the rural and regional populations in mental health outcomes.

Research has highlighted associations of individual factors such as sex, age, socioeconomic status, education, marital status, and body mass index (BMI) with likelihood of mental disorders, psychological distress and suicide in rural areas both in Australia and internationally [24–27]. The Australian Rural Mental Health Study was conducted in rural NSW between 2006–2012 and identified that personal and experiential factors, such as marital status and social support, were more relevant to mental health outcomes than factors relating to geographical location [15]. One study found that the highest prevalence of mental disorder was observed in middle-aged adults, but found no gender based differences [9]. Another study found that middle-aged women reported higher rates of mental illness compared to their metropolitan counterparts, while younger males had higher rates of suicide [10]. Additional research to identify the groups at risk of mental disorders, at a population level, is vital to develop suitable intervention and prevention strategies as well as access to appropriate services.

The Crossroads II study was a large-scale, cross-sectional study undertaken in the Goulburn Valley in rural Victoria between 2016–2018 [28] to investigate various aspects of health, lifestyle, diet, health attitudes and changes over time. Using the mental health outcomes data, this study sought to determine the prevalence of depression, and psychological distress and identify socio-demographic and lifestyle characteristics of individuals with poor mental health.

## Methods

### Ethics

Ethics approval was granted by the Goulburn Valley Health Ethics committee in in May 2016 (GVH-20/16). Written informed consent was obtained from all participants after information related to the study was provided. Separate consent was gained for the clinical assessment study [28]. The study was conducted in accordance with the tenets of the Declaration of Helsinki.

### Study setting

The Goulburn Valley is located 100-300 km away from Melbourne, the capital city of Victoria, Australia. The region was selected for study due to its shortage of health professionals and services as well as poor health outcomes. The Goulburn Valley is socioeconomically and demographically diverse, with a mix of First Nation, migrant and settled populations. The surveyed region included the population of Shepparton/Mooroopna (regional towns) and three surrounding smaller towns (Benalla to the east, Cobram to the north, and Seymour to the south).

### Population and study design

Data were collected by randomly selecting households and visiting each house to conduct a face-to-face questionnaire. Participants were included in the household survey if they were aged 16 years and over, but only those over the age of 18 were invited for clinical measurements and mental health assessment. To maximise participation, repeated visits were made during work hours, evenings, and weekends. The response rate was 62.7% (*n* = 1895) from 3022 households that were invited to participate in the survey [28, 29]. Subsequently, a randomly selected member of the household was invited to a 2-h screening clinic where screening measures were obtained (such as height and weight) and a further series of questionnaires were asked, including mental health tools. The mental health assessment was only conducted in Crossroads II. Response rate to the screening clinics was 48.1% (*n* = 741). Data for this study was collected by trained research assistants and entered into REDCap (Research Electronic Data Capture, Vanderbilt University, US) using iPads [28].

### Dependent variables

At the clinic, participants completed a series of self-administered mental health screening measures including Patient Health Questionnaire-9 (PHQ-9) [30] and the Kessler Psychological Distress Scale (K-10) [31]. These are the dependent variables for this analysis.

*The Kessler Psychological Distress Scale (K-10)* consists of 10-items on a five-point Likert scale, where a score of one indicates a response “none of the time”, and a score of five represents a response of “all of the time”. The sum of the scores range from 10 (no distress) to 50 (severe psychological distress) [32]. The K-10 survey is used to screen for psychological distress present in the most recent four weeks and has been found to have high precision in the 90^th^ to 99^th^ percentile range of the population [31]. The instrument effectively discriminates between cases and non-cases of mental disorders in the community according to Diagnostic and Statistical Manual of Mental Disorders-IV (DSM-IV) [33]. The K-10 survey is a validated tool that has been widely used in previous research in rural Australian populations [9, 15, 19].

*Patient Health Questionnaire-9 (PHQ-9)* is a nine-item questionnaire used to identify and assess severity of depression. It is based on the DSM-IV criteria for diagnosing major depressive disorders (MDD) in patients with medical illnesses. The instrument was reported to have a high test–retest reliability for psychiatric disorders (0.85) and high sensitivity and specificity (84 and 97% respectively) [34]. Questions refer to symptoms experienced in the previous two weeks. Scoring of this instrument indicates the level of depression, with 0–4 suggesting ‘no depression’, 5–9 ‘mild depression’, 10–14 ‘moderate depression’, 15–19 ‘moderate to severe depression’, and 20–27 ‘severe depression. The PHQ-9 is validated tool [32, 35] and has been used in previous research to measure depression in rural Australian populations [17].

For analysis, both K-10 and PHQ-9 scores were recategorized into binary outcomes in the regression model reported below. Depression was indicated by a PHQ-9 score of ≥ 10, and psychological distress was indicated by a K-10 score ≥ 21, reflecting threshold levels of psychological distress. The PHQ-9 cut off used in this study has been shown to have a sensitivity of 88% and a specificity of 88% [30] for diagnosis of major depressive disorder and while there is no universally agreed categories for K-10 scores, we used a cut off suggested based on the distribution and as recommended by the Australian Bureau of Statistics [36]. The K-10 cut off score of 21 was shown to have a sensitivity and specificity of 0.60 and 0.94, respectively, for identifying people who met the criteria for any current anxiety and depression [37].

### Independent variables: socio-demographic, lifestyle, and health characteristics

Demographic variables included age, sex, location, employment status, marital status, migrant status, Aboriginal and Torres Strait Islander status, European descent and education level. Age in years was recategorized and stratified into three categories for ease of reporting: ≤ 34, 35–54, and ≥ 55 years. Sex was coded as male or female and none was non-binary. Employment status was coded as full time, part time and unemployed/student. Education level was coded based on highest level attained: secondary education or lower, trade (certificates, vocational, diploma), and university or postgraduate education. Marital status was categorized as married (including de facto) and unmarried (divorced/widowed/separated and never married). Location was divided into regional centre (Shepparton and Mooroopna) and smaller towns (Benalla, Cobram, and Seymour) based on the town they resided in. Participants were categorised based on if they earned income (yes/no), and what their main source of income was including whether they were business owners, pensioners or dependent on their superannuation, and if they were salary earners (wages or salary/others).

Lifestyle factors included alcohol consumption (categorised as no alcohol intake, low risk: ≤ 4 drinks/day, or high risk: > 4 drinks/day) based on the Australian guidelines for alcohol consumption [20]. Smoking categorised current smokers or non-smoker (includes previous smokers). BMI was categorised as ‘underweight/normal weight’ (≤ 24.9 kg/m^2^), overweight (25–29.9 kg/m^2^), and obese (≥ 30 kg/m^2^). Participants were asked if they would consider themselves physically active (Yes/No), and if ‘Yes’, their reported average length of time (in minutes) per exercise session each day was used to derive the categories (adequate: at least 30 min and inadequate: < 30 min) [38].

Community factors included community participation (whether participants belonged to a club or group outside of work), and the time spent per month which was dichotomised into two categories: ≥ 10 h, < 10 h. Participants were also asked to rate their general health as either excellent, very good, good, fair, and poor, but for analysis this was grouped into excellent/very good and good/fair/poor due to the small counts in the latter three categories. Health condition variables were derived from a question in the household survey asking if participants have ever suffered from or are currently being treated for a range of chronic illnesses. These self-reported responses included diabetes, asthma, chronic obstructive pulmonary disease (COPD), and stroke.

### Statistical analysis

Descriptive analyses were used to determine each category's frequency of observations (n) and percentages (%). Cross tabulations were generated to describe the prevalence of major depression and psychological distress for each independent variable. The statistical significances for the cross tabulations were tested using the chi-squared test. The associations were further tested by odds ratios (OR) using bivariate logistic analyses for the two mental health outcomes. Those variables with *P* < 0.02 in the unadjusted analysis were retained and used in the multiple logistic regression analyses. Hence, multiple logistic regressions were examined by eliminating the non-significant variables to determine factors associated with psychological distress and major depression. STATA/MP version 17 (Stata Corp, College Station, TX, USA) was used to perform all statistical analyses. For the multiple linear regression analyses, a four-staged hierarchical modelling technique was conducted. The first stage (Model 1) included demographic factors of age group, sex, location, employment status, highest education attained, marital status, ethnicity, aboriginal status, income factors. In Model 2, lifestyle factors including body mass index (BMI), smoking status, alcohol consumption, physical activity and community participation were added to Model 1. In Model 3A, health factors including diabetes, asthma, stroke, heart disease, emphysema were added to Model 2 while Model 3B was similar to Model 3A, except that health factors were replaced with self-rated health.

## Results

### Sample population

The sociodemographic and health characteristics of 741 clinic participants who were included in the final analysis are presented in Table 1 (for 7 there was incomplete data). More than half lived in the smaller, rural towns (53.9%), just over half identified as female (55.6%), and most were aged 55 years or older (67.4%). Most of these participants (85.3%) were born in Australia and 36.7% were either divorced, widowed, or separated. For 48.0% of participants, secondary education or lower was their highest qualification. Further, superannuation was the main source of income for 44.9% participants. Few participants (1.9%) reported identifying as Aboriginal and/or Torres Strait Islander. Of lifestyle factors, about twelve percent consumed > 4 drinks/day and more than 69.3% were either overweight or obese. At the time of this study, majority of the participants (90.0%) were non-smokers.

**Table 1:** Sociodemographic and health characteristics of the study sample in Crossroads II (2016–18, *n* = 741)

**Variables**	**Frequency (%)**
**Demographics**	
**Age group in years**	
≤ 34	79 (10.7)
35–54	161 (21.9)
≥ 55	496 (67.4)
**Sex**	
Male	329 (44.4)
Female	412 (55.6)
Others	
**Location**^b^	
Rural	399 (53.9)
Regional	342 (46.2)
**Employment Status**	
Working full time	214 (43.0)
Working part time	130 (26.1)
Unemployed	154 (30.9)
**Highest Education Attained**	
Completed secondary education or less	355 (48.0)
Completed trade/certificate/diploma	211 (28.5)
Completed university	174 (23.5)
**Marital Status**	
Married/De-facto	450 (63.3)
Unmarried^a^	261 (36.7)
**Ethnicity**	
Australian-born	631 (85.3)
non-Australian born	109 (14.7)
**Indigenous Status**	
Aboriginal and/or Torres Strait Islander	
No	727 (98.1)
Yes	14 (1.9)
**Income factors**	
**Earns income**	
Yes	512 (77.7)
No	147 (22.3)
**Main source of income**	
*Main source of income*	
Wages or salary/others	306 (43.6)
Pension/superannuation	315 (44.9)
Own business/investment	81 (11.5)
**Lifestyle factors**	
**BMI**	
Underweight/Healthy Weight (≤ 24.9)	222 (30.7)
Overweight	265 (36.7)
Obese (≥ 30)	236 (32.6)
**Smoking**	
Non-smoker	641 (90.0)
Current smoker	71 (10.0)
**Alcohol Consumption (yes/no)**	
None	151 (20.4)
< 4 drinks	501 (67.6)
4 + drinks	89 (12.0)
**Physical activity**	
None	191 (26.8)
Inadequate	177 (24.9)
Adequate	344 (48.3)
Community Participation	
No	285 (40.0)
Yes	427 (60.0)
Time spent/month in the community group	
> 10 h	272 (63.6)
≤ 10 h	156 (36.4)
**Health factors**	
Diabetes status	
No	646 (87.2)
Yes	95 (12.8)
Asthma	
No	608 (82.1)
Yes	133 (18.0)
Emphysema	
No	724 (97.7)
Yes	17 (2.3)
Stroke	
No	28 (3.8)
Yes	28 (3.8)
**Self-rated general health**	
Very good/excellent	345 (46.6)
Poor/good	396 (53.4)
**Mental health status**	
**K-10 scores—Psychological distress**	
Low (10–15)	431 (66.5)
Moderate (16–21)	112 (17.3)
High (22–29)	71 (11.0)
Very high (30 +)	34 (5.3)
**PhQ-9 scores—Depression**	
None (0–4)	456 (65.1)
Mild (5–9)	150 (21.4)
Moderate (10–14)	58 (8.3)
Moderate-severe (15–19)	24 (3.4)
Severe (20–27)	13 (1.8)

*BMI* Body mass index

^a^widowed, divorced, separated, and never married

^b^Rural include Shepperton and Mooroopna towns while regional included Benalla, Cobram, and Seymour towns

This survey included data from 133 participants (18.0%) with asthma and 95 with diabetes (12.8%). Most participants perceived their general health as either poor, fair or good (53.4%) while 46.6% perceived their health as either very good or excellent.

### Prevalence of threshold-level K-10 psychological distress and PHQ-9 depression

The overall prevalence of mental health problems was 13.6% and 16.2% for threshold-level depression (PHQ-9 ≥ 10) and psychological distress (K-10 ≥ 21), respectively. This translates to one in six participants who showed signs of psychological distress whereas approximately one in every seven participants indicated a score consistent with major depression, most of whom were categorised as moderate PHQ-9 depression (8.3%, Table 1). Of those with threshold levels of K-10 scores, 19.0% and 10.5% had seen a psychologist or a psychiatrist respectively in the past year while 14.3% were taking medications for anxiety. Among those with threshold-level depression, 24.2% and 9.5% had seen a psychologist or a psychiatrist, respectively in the past year, while 42.1% were taking anxiety medications at the time of this study.

#### Factors associated with psychological distress: unadjusted analysis

Table 2 presents the prevalence, unadjusted odd ratios and 95% confidence interval (CI) for factors associated with psychological distress. A total of 648 participants completed the K-10 survey. Higher rates of threshold level psychological distress were detected among current smokers (37.5%), participants who consumed more than 4 standard drinks per day (32.9%), those aged 18 – 34 years (27.4%), those who had obesity (26.3%), and/or were living with asthma (26.0%) or COPD (25.0%) at the time of this study. Among the 11 Aboriginal and Torres Strait Islander participants in this study, 5 reported threshold-level psychological distress. Of all participants who self-rated their ‘general health’ as ‘poor or good’, about one fourth (23.5%) experienced threshold-level psychological distress (Table 2). In the unadjusted analysis, we found that older age, higher education, owning a business/investment, less alcohol, physical activity, and community participation were associated with lower K-10 scores, while high K-10 scores were associated with being unmarried, identifying as Aboriginal and/or Torres Strait Islander, a lack of income, smoking, obesity, living with asthma and poorer self-rated general health (Table 2).

**Table 2:** Prevalence and unadjusted odd ratio (OR) of factors associated with self-reported mental health problems of psychological distress (*n* = 648) and depression (*n* = 701) among rural/regional Victorians in Crossroads II study (2016–18)

**Demographics**	**Psychological distress**	**OR [95%CI]**	**Depression**	**OR [95%CI]**
**Age group in years**				
≤ 34	20 (27.4)	Ref	17 (21.8)	Ref
35–54	28 (19.6)	0.65 [0.33, 1.25]	31 (19.9)	0.89 [0.46, 1.73]
≥ 55	57 (13.4)	**0.41 [0.23, 0.73]**	47 (10.2)	**0.41 [0.22, 0.75]**
**Sex**				
Male	41 (14.5)	Ref	40 (12.9)	Ref
Female	64 (17.5)	1.26 [0.82, 1.92]	55 (14.1)	1.11 [0.72, 1.72]
**Location**^a^				
Rural	52 (14.1)	Ref	47 (12.7)	Ref
Regional	53 (18.9)	1.42 [0.93, 2.16]	48 (14.6)	1.17 [0.76, 1.81]
**Employment Status**				
Working full time	32 (15.5)	Ref	25 (12.0)	Ref
Working part time	13 (10.4)	0.63 [0.32, 1.26]	15 (12.0)	1.00 [0.51, 1.99]
Unemployed	30 (20.4)	1.41 [0.82, 2.45]	25 (17.1)	1.51 [0.83, 2.75]
**Highest Education Attained**				
Completed secondary education or less	63 (20.6)	Ref	47 (14.3)	Ref
Completed trade/certificate/diploma	26 (13.8)	0.62 [0.37, 1.01]	33 (16.4)	1.17 [0.72, 1.91]
Completed university	16 (10.5)	**0.45 [0.25, 0.81]**	15 (8.7)	0.57 [0.31, 1.05]
**Marital Status**				
Married/De-facto	51 (12.7)	Ref	39 (9.1)	Ref
Unmarried	50 (22.1)	**1.96 [1.27, 3.01]**	52 (21.2)	**2.71 [1.73, 4.25]**
**Ethnicity**				
Australian-born	85 (15.5)	Ref	82 (13.8)	Ref
non-Australian born	20 (20.6)	1.42 [0.83, 2.45]	13 (12.5)	0.90 [0.48, 1.67]
**Indigenous Status**				
Aboriginal and/or Torres Strait Islander				
No	100 (15.7)	Ref	90 (13.0)	Ref
Yes	5 (45.5)	**4.48 [1.34, 14.95]**	5 (45.5)	**5.56 [1.66, 18.58]**
**Income factors**				
**Earns income**				
Yes	72 (14.6)	Ref	64 (13.0)	Ref
No	32 (22.2)	**1.67 [1.05, 2.66]**	25 (17.1)	1.38 [0.83, 2.29]
*Main source of income*				
Wages or salary/others	44 (15.8)	Ref	41 (13.6)	Ref
Pension/superannuation	52 (18.8)	1.23 [0.79, 1.92]	45 (15.2)	1.13 [0.72, 1.79]
Own business/investment	4 (5.6)	**0.31 [0.11, 0.91]**	5 (6.3)	0.43 [0.16, 1.12]
**Lifestyle factors**				
**BMI,** kg/m2				
Underweight/Healthy Weight (≤ 24.9)	23 (11.7)	Ref	18 (8.7)	Ref
Overweight	23 (10.2)	0.86 [0.46, 1.58]	26 (10.4)	1.23 [0.65, 2.30]
Obese (≥ 30)	56 (26.3)	**2.68 [1.58, 4.56]**	26 (21.1)	**2.83 [1.59, 5.05]**
**Smoking**				
Non-smoker	77 (13.6)	Ref	72 (11.8)	Ref
Current smoker	24 (37.5)	**3.80 [2.17, 6.66]**	19 (29.2)	**3.10 [1.72, 5.58]**
**Alcohol Consumption (yes/no)**				
None	28 (23.5)	Ref	24 (18.9)	Ref
< 4 drinks	50 (11.2)	**0.41 [0.24, 0.69]**	48 (9.9)	**0.47 [0.28, 0.80]**
4 + drinks	27 (32.9)	1.60 [0.85, 2.98]	23 (26.1)	1.52 [0.79, 2.91]
**Physical activity**				
None	38 (23.2)	Ref	39 (21.6)	Ref
Inadequate	26 (16.7)	0.66 [0.38, 1.16]	23 (13.7)	0.58 [0.33, 1.02]
Adequate	37 (12.0)	**0.45 [0.27, 0.74]**	29 (8.8)	**0.35 [0.21, 0.59]**
Community Participation				
No	58 (22.9)	Ref	53 (19.6)	Ref
Yes	43 (11.4)	**0.43 [0.28, 0.67]**	38 (9.4)	**0.42 [0.27, 0.67]**
Time spent/month in the community group				
> 10 h	18 (7.5)	Ref	21 (8.1)	Ref
≤ 10 h	25 (18.1)	**2.72 [1.42, 5.19]**	17 (11.6)	1.49 [0.76, 2.92]
**Health factors**				
Diabetes status				
No	92 (16.1)	Ref	83 (13.5)	Ref
Yes	13 (16.9)	1.06 [0.56, 2.00]	12 (13.6)	1.01 [0.53, 1.93]
Asthma				
No	78 (14.3)	Ref	70 (12.1)	Ref
Yes	27 (26.0)	**2.09 [1.27, 3.45]**	25 (20.7)	**1.90 [1.14, 3.15]**
Emphysema				
No	101 (16.0)	Ref	90 (13.1)	Ref
Yes	4 (25.0)	1.75 [0.55, 5.54]	5 (33.3)	**3.31 [1.11, 9.91]**
Stroke				
No	102 (16.4)	Ref	93 (13.8)	Ref
Yes	3 (12.5)	0.73 [0.21, 2.50]	2 (8.0)	0.55 [0.13, 2.35]
Heart disease				
No	3 (21.4)	Ref	70 (12.1)	Ref
Yes	9 (16.7)	1.42 [0.39, 5.19]	12 (13.6)	1.18 [0.23, 6.06]
**Self-rated general health**^a^				
Very good/excellent	25 (8.1)	Ref	17 (5.1)	Ref
Poor/good	80 (23.5)	**3.46 [2.14, 5.59]**	78 (21.2)	**5.00 [2.89, 8.65]**

*CI* Confidence intervals* of the odd ratios are shown in parenthesis*, *BMI* Body mass index

^a^Regional include Shepperton and Mooroopna towns while rural included Benalla, Cobram, and Seymour towns.* Significant variables are bolded*. Psychological distress was derived from K−10 questionnaire, depression was derived from PhQ−9 questionnaire

#### Factors associated with major depression: unadjusted analysis

Table 2 also presents the prevalence, unadjusted odd ratios and 95% CIs for factors associated with depression. Responses were recorded from 701 participants who completed the PHQ-9 survey. Participants who identified as Aboriginal and/or Torres Strait Islander (*n* = 11), those living with emphysema (*n* = 15) and current smokers (*n* = 65) had the highest prevalence of depression (45.5%, 33.3% and 29.2%, respectively). In addition, individuals aged 18-34 years (21.8%), those with obesity (21.1%, each) had a high prevalence of depression in this study. Compared with married/de facto persons, unmarried individuals had a higher prevalence of depression. Similar to psychological distress, 21.2% of those who self-rated their ‘general health’ as ‘poor, fair or good’ experienced major depression (Table 2).

Additional information on the unadjusted odd ratios of factors associated with major depression in different subgroups were also shown in Table 2. Older age, less drinking, physical activity, and community participation were associated with lower likelihood of depression, whereas identifying as Aboriginal and/or Torres Strait Islander, smoking, obesity, being unmarried and presence of asthma, or emphysema, and poor to good self-rated health, were associated with higher likelihood of depression (Table 2). There was no association between location and both mental health outcomes in the unadjusted analysis.

#### Factors associated with threshold-level K-10 psychological distress and PHQ-9 depression in rural Victoria

The results of the final model from the hierarchal multivariable analysis are presented in Table 3 for threshold level psychological distress and depression. However, those obtained for the different stages of the modelling are presented in Supplementary Tables (S-Table 1 and S-Table 2) respectively. In the first model (Model 1) which adjusted for the demographic variables, part time work was associated with lower K-10 scores while not being married was associated with higher K-10 scores (S-Table 1). The likelihood of experiencing psychological distress was reduced by half in those who participated in their communities compared to those who did not, and this was consistent after adjusting for the demographic, lifestyle, and health factors. Of lifestyle factors, being a current smoker increased the likelihood of experiencing threshold level psychological distress by more than 3 folds and this was consistent across the Models. Obesity was associated with a twofold increase in the risk of psychological distress after adjusting for all the potential confounding factors (Model 3A: aOR 2.72 95%CI 1.23, 6.03). Although participants who identified as Aboriginal and/or Torres Strait Islander had a higher odd of psychological distress (S-Table 1), this was not statistically significant after adjusting for all the potential confounders in Table 3.

**Table 3:** Adjusted odd ratio (aOR) of factors associated with threshold level distress and depression among rural/regional Victorians in Crossroads II study (2016–18)

**Variables**	**Model steps**	**Psychological distress**		**Depression**	
**Demographics**	Model 1	**Final Model 3A**	**Final Model 3B**	**Final Model 3A**	**Final Model 3B**
**Age group in years**	Model 1				
≤ 34		*Ref*	*Ref*	*Ref*	*Ref*
35–54		0.50 [0.17, 1.43]	0.45 [0.16, 1.31]	1.02 [0.34, 3.04]	0.85 [0.29, 2.59]
≥ 55		0.70 [0.24, 2.06]	0.54 [0.18, 1.55]	0.63 [0.19, 2.04]	0.45 [0.14, 1.46]
**Sex**	Model 1				
Male		*Ref*	*Ref*	*Ref*	*Ref*
Female		1.39 [0.71, 2.71]	1.65 [0.85, 3.20]	0.94 [0.47, 1.89]	1.13 [0.57, 2.24]
Others					
**Location**^a^	Model 1				
Rural		*Ref*	*Ref*	*Ref*	*Ref*
Regional		1.69 [0.89, 3.19]	1.60 [0.85, 3.00]	1.82 [0.92, 3.60]	1.74 [0.88, 3.45]
**Employment Status**	Model 1				
Working full time		*Ref*	*Ref*	*Ref*	*Ref*
Working part time		0.48 [0.21, 1.12]	0.53 [0.23, 1.22]	1.01 [0.43, 2.37]	1.17 [0.49, 2.78]
Unemployed		0.95 [0.45, 1.97]	0.98 [0.47, 2.05]	1.27 [0.57, 2.85]	1.53 [0.67, 3.45]
**Highest Education Attained**	Model 1				
Completed secondary education or less		*Ref*	*Ref*	*Ref*	*Ref*
Completed trade/certificate/diploma		0.90 [0.42, 1.90]	0.94 [0.45, 1.97]	1.70 [0.79, 3.63]	1.65 [0.77, 3.54]
Completed university		1.00 [0.44, 2.28]	1.15 [0.50, 2.65]	1.44 [0.57, 3.64]	1.87 [0.73, 4.81]
**Marital Status**	Model 1				
Married/De-facto		*Ref*	*Ref*	*Ref*	*Ref*
Unmarried		1.38 [0.71, 2.52]	1.28 [0.68, 2.40]	**2.75 [1.41, 5.37]**	**2.56 [1.30, 5.03]**
**Ethnicity**	Model 1				
Australian-born		*Ref*	*Ref*	Ref	Ref
non-Australian born		1.74 [0.79, 3.85]	1.71 [0.79, 3.72]	0.61 [0.23, 1.61]	0.63 [0.24, 1.65]
**Indigenous Status**	Model 1				
Aboriginal and/or Torres Strait Islander					
No		*Ref*	*Ref*	*Ref*	*Ref*
Yes		5.45 [0.96, 30.78]	4.73 [0.90, 24.9]	2.32 [0.37, 14.63]	2.26 [0.39, 13.20]
**Income factors**	Model 1				
**Earns income**	Model 1				
Yes		*Ref*	*Ref*	*Ref*	*Ref*
No		1.58 [0.57, 4.36]	1.58 [0.58, 4.35]	0.69 [0.22, 2.17]	0.72 [0.23, 2.27]
*Main source of income*	Model 1				
Wages or salary/others		*Ref*	*Ref*	*Ref*	*Ref*
Pension/superannuation		1.44 [0.65, 3.16]	1.54 [0.71, 3.33]	1.38 [0.60, 3.17]	1.41 [0.62, 3.21]
Own business/investment		0.62 [0.15, 2.55]	0.70 [0.17, 2.83]	0.68 [0.16, 2.89]	0.75 [0.17, 3.21]
**Lifestyle factors**	Model 2				
**BMI,** kg/m2	Model 2				
Underweight/Healthy Weight (≤ 24.9)		*Ref*	*Ref*	*Ref*	*Ref*
Overweight		1.18 [0.50, 2.78]	1.17 [0.50, 2.76]	2.44 [0.89, 6.68]	2.40 [0.88, 6.56]
Obese (≥ 30)		**2.72 [1.23, 6.03]**	**2.33 [1.05, 5.15]**	**3.79 [1.42, 10.1]**	**2.96 [1.14, 7.70]**
**Smoking**	Model 2				
Non-smoker		*Ref*	*Ref*	*Ref*	*Ref*
Current smoker		**3.55 [1.47, 8.56]**	**3.25 [1.34, 7.88]**	1.55 [0.60, 3.99]	1.36 [0.52, 3.48]
**Alcohol Consumption (yes/no)**	Model 2				
None		*Ref*	*Ref*	*Ref*	*Ref*
< 4 drinks		0.61 [0.28, 1.31]	0.64 [0.30, 1.37]	0.55 [0.24, 1.28]	0.62 [0.27, 1.44]
4 + drinks		0.91 [0.31, 2.62]	1.03 [0.36, 2.99]	0.81 [0.27, 2.44]	1.04 [0.34, 3.14]
**Physical activity**	Model 2				
None		*Ref*	*Ref*	*Ref*	*Ref*
Inadequate		0.72 [0.32, 1.61]	0.78 [0.35, 1.70]	0.78 [0.35, 1.78]	0.81 [0.36, 1.81]
Adequate		0.59 [0.28, 1.24]	0.62 [0.30, 1.29]	**0.42 [0.19, 0.92]**	**0.42 [0.19, 0.93]**
Community Participation	Model 2				
No		*Ref*	*Ref*	*Ref*	*Ref*
Yes		**0.52 [0.28, 0.98]**	0.54 [0.29, 1.01]	0.51 [0.26, 1.03]	0.52 [0.26, 1.02]
**Health factors**					
Diabetes status	Model 3A				
No		*Ref*		*Ref*	
Yes		0.59 [0.18, 1.93]		0.82 [0.27, 2.47]	
Asthma	Model 3A				
No		*Ref*		*Ref*	
Yes		1.66 [0.75, 3.65]		1.32 [0.56, 3.14]	
Emphysema	Model 3A				
No		*Ref*		*Ref*	
Yes		1.86 [0.32, 10.8]		4.15 [0.61, 28.17]	
Stroke	Model 3A				
No		*Ref*		*Ref*	
Yes		1.07 [0.19, 13.6]		0.39 [0.04, 4.03]	
Heart disease	Model 3A				
No		*Ref*		*Ref*	
Yes		1.63 [0.20, 13.6]		2.02 [0.22, 18.28]	
**Self-rated general health**^a^	Model 3B				
Very good/excellent			*Ref*		*Ref*
Poor/good			**2.18 [1.06, 4.47]**		**3.58 [1.54, 8.34]**

*CI* Confidence intervals *of the odd ratios are shown in parenthesis*, *BMI* Body mass index

^a^Regional include Shepperton and Mooroopna towns while rural included Benalla, Cobram, and Seymour towns. *Significant variables are bolded.* Empty cells are variables not included in the specific model. Psychological distress was derived from K−10 questionnaire, depression was derived from PhQ−9 questionnaire

The factors associated with higher likelihood of mental health outcomes of depression included regional residence, not being married, obesity, poorer self-rated health while community participation and more strongly, being physically active (Table 3) were associated with lower likelihood of depression. As presented in the final models (Model 3A & B), compared with those who did not get involved in any physical activity, participants who reported adequate physical activity were 60% less likely to experience threshold-level depression. However, the significant reduction in the threshold-level depression in people who belonged to a community group found in Model 2 (S-Table 2), was no longer significant when the health conditions of the participants were considered in Models 3A and B (Table 3). Those who lived in regional towns were twice more likely to experience threshold-level depression compared to their counterparts in the rural towns after adjusting for other demographic variables in Model 1 (S-Table 2), but this association became non-statistically significant when community participation and health conditions of the participants were considered in Models 2 and 3.

After adjusting for the potential confounders in Table 3, none of the individual health conditions were associated with mental health problems, but those who self-rated their health as ‘poor, fair or good’ were twice (95%CI 1.06, 4.47) and four times (95%CI 1.54, 8.34) more likely to experience threshold-levels of psychological distress and depression, respectively.

## Discussion

With rising rates of mental health disorders across Australia, we sought to examine the prevalence and factors associated with mental health conditions in four regional/rural communities. Using two recognised psychological diagnostic tools, we were able to investigate the prevalence of mental health conditions with demographic, health, and lifestyle data. The study found a higher prevalence of psychological distress and depression in this rural population which reflected other rural studies [15, 19]. Personal and lifestyle factors such as obesity, smoking and community participation were more relevant to mental health outcomes than degree of rurality in Victoria; the latter of which showed variation in outcomes that was dependent on the individuals’ involvement in community groups or their health condition. It is worth noting that participants of the K-10 and PHQ-9 surveys who reported threshold-levels of distress or depression may not have had a known diagnosis of a mental health condition. This suggests there may be undiagnosed or under-recognised mental health conditions across the region.

This study found significant rural vs regional differences in depression after adjusting for the other demographic variables. This association became non-statistically significant when the individuals’ involvement in the community and their health status were considered. Previous studies have acknowledged that differences between rural and urban areas are more complex than geographical distance, given the large variability in socio-demographic and lifestyle factors [11, 13]. Rural populations are known to experience disadvantage compared to metropolitan regions due to a variety of factors including lower incomes, less employment opportunities, and poorer access to health services, education, and transport [39]. Despite this, some studies have reported similar prevalence of mental illness between rural and urban areas [10–13], which could relate to our finding of no significant location differences in the unadjusted analysis (Table 2). The findings from this study add to the growing evidence that factors relating to geographical location has some impact on mental health problems but not as much as personal and experiential factors.

The overall prevalence of psychological distress in our study is comparable to the 20% reported across the nation [40] and consistent with multiple national surveys that have shown a substantially higher prevalence of psychological distress amongst Aboriginal and/or Torres Strait Islanders [41, 42] compared to non-Indigenous people. Despite low numbers, our study found that participants of Aboriginal and Torres Strait Islander origin were, on average, twice as likely to report major depression and seven times more likely to report psychological distress compared to the others. While this suggests very high rates of distress and depression in the Aboriginal and Torres Strait Islander population, the cultural appropriateness of these tools in non-Western cultures and the low participation rate of the group in this study, should be considered.

Several lifestyle factors are also significantly associated with mental illness, including smoking, and obesity. Those who currently smoke were three times more likely to report major depression, and four times more likely to report psychological distress than those who do not smoke. Similarly, those who had obesity had a threefold increase in psychological distress and a fourfold increase in depression than those with a lower BMI. However, participating in physical activity reduced the likelihood of threshold-level depression by about 60% after adjusting for all other variables except for their health conditions. Both smoking and obesity have been associated with poor mental health within the general population, with previous research suggesting a bi-directional relationship between mental health and each factor [43, 44]. In a recent review study [45], physical activity was about 1.5 times more effective at reducing mental health problems than counselling or the leading medications, and the largest benefit was seen in people with depression. The present findings affirm that these results are applicable to a rural Australian population and support the need for community targeted lifestyle health promotion that engages and enables people to develop positive lifestyle changes.

In this study, we found that belonging to a community group appears to have significant benefit to people’s mental health condition, but not working (pensioners/retirees) had a negative impact on mental health condition, as reported in a previous study [18]. While poorer mental health in people who are not working has been attributed to both the impact of unemployment and existing mental health problems, the finding that any amount of community participation appeared beneficial may mitigate this effect if people participate in community activities. Previous studies have also highlighted the importance of social factors, such as social interconnectedness, access to services, and perception of community on the mental health of rural Australians [11, 15, 19]. In 2018, the Victorian Department of Health reported that rural Victorian dwellers were more likely to participate in community groups than their metropolitan neighbours [46]. This seems to assist in reducing the prevalence of mental illness, although better mental health may also enable participation.

It is important to note that demographic factors, such as not living with a partner, was associated with a increase in the odds of mental health outcomes while working part-time reduced the odds of mental health problems in this study. However, the lifestyle factors mentioned previously had a stronger effect on mental health than these demographic factors. In the unadjusted analysis, we found lower risk of mental health problems among older people. Although this effect was no longer significant after adjusting for the potential confounders, previous studies [9, 10, 47], including the 2007 National Survey of Mental Health and Wellbeing [48], have identified lower rates of mental illness in the older population. An increase in prevalence of psychological distress with age was reported previously [9, 49], with a systematic review showing a lower pooled prevalence of major depression (7.2%) in older Australians (75 years and older) [50]. Among older Australians (> 60 years), another study found 8.2% prevalence of depression [51] which was similar to the prevalence found among the ≥ 55 years age group (10.2%) in this study. It is important to note that most of these studies recruited only older participants.

In this study, poorer perceived general health was progressively associated with higher odds for mental health outcomes after adjusting for all potential confounders. This is in agreement with previous report in Australia which showed that separate global ratings of poorer health were associated with higher threshold mental health problems [15, 19].

### Strengths and limitations

This study examined a wide range of socio-demographic, lifestyle, and health factors associated with mental health problems in rural Victoria. Identifying relevant factors associated with mental illness can aid in the delivery of targeted and effective mental health screening and treatment services in the region to stem the rising prevalence of mental health conditions nationwide. The study also had a high response rate (65%) relative to other similar studies in rural areas [14, 15] and had a relatively even distribution of responses from the regional centre and smaller towns. It was able to capture mental health data from a variety of sources including validated scales, symptoms, medications, and mental health service utilization. The study also provides deeper insights into the mental health status of a rural population by collecting data that provides an excellent basis for understanding health care needs for this population. Despite these strengths, the study has some limitations to consider. All participants in this study resided within the town boundaries and the sample did not include people living in smaller 'hamlets', rural properties, or on farms. Rural Australian populations are not homogenous and experience large differences in sociodemographic factors between communities [52]. It is thus difficult to generalise these results to all regional and rural Australian populations. The study was also limited by lower response numbers to the K-10 and PHQ-9 scales. Additionally, there is a possibility of undiagnosed or under- recognised mental health conditions in this study, since PHQ9 and K-10 are looking at different time periods. The use of self-reported data is also a limiting factor as it can lead to over-reporting or under-reporting of mental illness, but this was supported by the validated scales used in this study. As this is a cross-sectional study, it is unable to identify causal relationships between variables, or examine trends over time. The higher prevalence of psychological distress reported in the current study should be interpreted with caution since the use of a lower classification for K-10 scores may have lower positive predictive values for mental illness as reported in some other studies [37, 53].

## Conclusion

This study found the expected prevalence of mental health risk among rural Victorians, with reports of psychological distress observed in about one sixth of the respondents. Personal and lifestyle factors, especially smoking, obesity and Aboriginal status were more relevant to mental health outcomes than degree of rurality in Victoria. Factors such as not living with a partner, smoking and obesity, were significantly associated with a higher prevalence of mental health problems, while community participation and physical activity appeared to be protective against mental health risk in this study. Therefore, promotion of lifestyle interventions and community activities are urgently needed to improve mental health and wellbeing in rural populations. Interventions tailored to the rural context, community needs, and the available mental health workforce could assist in reducing the burden of mental illness, and may include general practitioners, face-to-face services wherever possible (with a focus on prevention and early intervention), supplemented by specialised services delivered by telehealth.

## Supplementary Information

**Additional file 1: Table S1.** Summary of availableliterature on mental health symptoms in rural and urban regions in Australia. **Table S2.** Hierarchical modelling showing adjusted odd ratio(aOR) of factors associated with threshold level distress among rural/regional Victorians in Crossroads II study (2016-18, *n*=741). **Table S3.** Hierarchical modelling showing adjusted odd ratio(aOR) of factors associated with threshold-level depression using PHQ-9 among rural/regional Victorians in Crossroads II study (2016-18, *n*=701).

## Data Availability

The dataset supporting the conclusions of this article is included within the article (and its additional files). Data is also available on request from the corresponding author D.S.

## Abbreviations

MH: Mental health
K-10: Kessler Psychological Distress Scale
PHQ-9: Patient Health Questionnaire-9
BMI: Body mass index
NSW: New South Wales
AOR: Adjusted odds ratio
CI: Confidence interval
