# Supplementary material for: Prevalence and factors associated with mental health problems of psychological distress and depression among rural Victorians – analysis of cross-sectional data (Crossroads II)
Source: BMC Psychiatry. 2023 Jun 20;23:450. doi: 10.1186/s12888-023-04931-5 (PMC10283169; doi:10.1186/s12888-023-04931-5)
Supplement: Supplementary file 1 — Additional file 1: Table S1. Summary of availableliterature on mental health symptoms in rural and urban regions in Australia. Table S2. Hierarchical modelling showing adjusted odd ratio(aOR) of factors associated with threshold level distress among rural/regional Victorians in Crossroads II study (2016-18, n=741). Table S3. Hierarchical modelling showing adjusted odd ratio(aOR) of factors associated with threshold-level depression using PHQ-9 among rural/regional Victorians in Crossroads II study (2016-18, n=701). [file 12888_2023_4931_MOESM1_ESM.docx]

Supplementary Tables

S1: Summary of available literature on mental health symptoms in rural and urban regions in Australia.

| Author (year) | Country | Urban or Rural | Sample size (n) | Type of Study | Aims | Variables | Limitations |
| --- | --- | --- | --- | --- | --- | --- | --- |
| Kilkkinen et al (2007) | Australia | Rural | N=1563 | Cross-sectional | To describe the prevalence of psychological distress, depression and anxiety in three Australian rural settings and to identify the levels of risk by gender and age | Independent:  Sociodemographic factors (age, sex)  Dependent:  Psychological distress (K-10 scale), anxiety and depression (HADS) | - the sample might be biased because of downward morbidity, whereby non-respondents are more likely to have symptoms - social desirability of self-reported mental health might have had an effect on the prevalence rates - cross sectional study |
| Caldwell et al (2004) | Australia | Rural | N= 10 641 | Cross-sectional | To compare the prevalence of mental health disorders and the use of professional help by area of residence, age and sex; and to determine whether the differences parallel differences in suicide rates | Independent:  Age, sex  Rurality (RRMA index)  Dependent  Suicide rates, affective and anxiety disorders (modified version of the Composite International Diagnostic Interview according to ICD 10), use of health professionals for mental health problems | - could not identify Indigenous status - cross sectional study - self-reported symptoms may be unreliable - small sample sizes in rural and remote areas compared to metropolitan areas - unable to investigate reasons for seeking or not seeking professional help |
| Murray et al (2004) | Australia | Rural | N=7615 | Cross-sectional | To investigate whether accessibility/remoteness correlates with mental health. | Independent:  Rurality (ARIA),  Control:  age; gender; highest level of education; employment; country of birth; indigenous heritage; and household composition  Dependent:  Psychological distress (K10 scale), negative affect (10-item Negative Affect), disability (SF-12), subjective wellbeing (Satisfaction With Life scale) | - Accessibility was measured only on ARIA, other variables may be more sensitive indicators of accessibility as an impact on mental health - Generalisation of subjects from extremely remote areas - Cross sectional study |
| Eckert et al (2004) | Australia | Both | N=2545 | Cross-sectional | To determine whether mental illness is associated with accessibility and remoteness. | Independent:  Rurality (ARIA)  Dependent:  Psychological distress (K10 scale), depression (SF-12)  Control:  Age, sex, marital status, country of birth, highest education attained, occupation, employment status, household income | - Residents with no telephone connection, people in hospitals, nursing homes, homeless people, or those in other major institutions were not included - Cross sectional study |
| Judd et al (2002) | Multiple | Both | 2 second generation studies  3 national household surveys | Systematic review | Critically review studies measuring prevalence in rural as opposed to urban location | Independent:  Sociodemographic and lifestyle characteristics (age, gender, education, household income, marital status, employment, social support, ethnicity, life events, alcohol problems)  Dependent:  Mental disorders according to | - Use of a ‘one size fits all’ approach for urban and rural definitions leads to an averaging out of differences that can be highly variable. Recommendation for future studies to better examine independent variables that can be risk factors for mental health disorders. |
| Lee and Browne (2008) | Australia | Rural | N=5391 | Cross-sectional | To ascertain the relationship between subjective well-being and sociodemographic factors, mental and physical health of a sample of rural residents | Independent:  Sociodemographic factors (age, gender, employment status, living circumstance)  Dependent:  Life satisfaction (satisfaction with life scale), psychological distress (K-10), physical and mental disability (SF-12) | - High non-response rate may cause non-response bias, whereby people with low levels of life satisfaction, physical disability, mental disability and high levels of psychological distress might have chosen not to participate - Cross-sectional study |
| Kelly et al (2011) | Australia | Rural | N=2,639 | Cross-sectional | To identify determinants of mental health and well-being within rural and remote communities | Independent:  Personality (EPI-12), Recent adverse life events/difficulties, Social support, Sense of community index, Infrastructure and services accessibility, Perception of drought severity, Duration of participants’ exposure to the specific rural environment and community, The Index of Relative Socio-Economic Disadvantage, District population change, Drought severity, Remoteness (ARIA and ASGC)  Dependent:  Psychological distress (K-10 scale), ability to perform everyday duties and tasks, overall physical health, overall mental health, satisfaction with relationships in general, and overall satisfaction with life | - Cross sectional study - Low response rate |
| Allen et al (2012) | Australia | Rural | N=4219 | Cross-sectional | To examine whether the association of psychosocial factors with psychological distress outcomes varies with increasing remoteness | Independent:  Sociodemographic characteristics (age, gender, education and marital status), social support, remoteness (ARIA)  Dependent:  Psychological distress (K-10 scale), | - younger people were not represented - does not take into account modern forms of socialisation such as instant video, chat and text messaging and social networking services, or the importance of persons outside the community for sustaining mental health - unable to assess effect of previous area of residence or migration - cross sectional study |
| Handley et al (2018) | Australia | Rural | N= 2639 | Longitudinal | To explore the contributions of a range of individual, social, and community factors to the trajectory of depressive symptoms among a cohort of rural and remote residents. | Independent  Sociodemographic data (age, gender, employment status, marital status, smoking status, unintentional injuries during the previous 12 months, perceived financial status, social integration and support, current alcohol use, neuroticism, concern about specific rural community characteristics, recent personal adverse life events)  Dependent  Depression (PHQ-9) | - help-seeking and service use patterns were not included in the analyses - low initial response rate and the under-representation of younger people initially recruited - differential loss to follow-up across the phases of the study, with higher attrition among those with depression, younger age groups, and those with a lower perceived financial position - some missing data within waves which, while mostly missing at random, may have influenced the outcomes of the models. |
| Butterworth et al (2014) | Australia | Rural | N= 2,639 | Cross-sectional | To investigate variations in the prevalence of significant psychological distress across regions, and quantifies the relative impact of family functioning and social support | Independent  Demographic factors (age, sex, partner status, chronic physical health conditions, educational attainment)  Interpersonal factors (social support, family conflict, quality of relationships)  Household and family factors (household income)  Remoteness (ASGC)  Dependent  Psychological distress (K-10) | - low initial response rate - potential sampling bias towards older people, those born in Australia and to homeowners - cross-sectional study |
| Kelly et al (2010) | Australia | Rural | N=2,639 | Longitudinal | To investigate the determinants and outcomes of common mental disorders in rural and remote communities, with specific reference to individual, family/household, and community factors (including environmental adversity) | Independent  Age, rurality (ASGC)  Dependent  Psychological distress (K-10) | - low initial response rate - potential sampling bias towards older people, those born in Australia and to homeowners |

^Abbreviations: RRMA = Rural, Remote and Metropolitan Area; HADS = Hospital Anxiety and Depression Scale; ARIA = Accessibility and Remoteness Index of Australia.^

^A literature review was conducted in January 2022 to identify studies that have investigated the prevalence of mental illness in non-metropolitan regions of Australia. A search was performed on PubMed with the following search terms: “prevalence”, “mental”, “psychiatric” “illness”, “disorder”, “psychological distress”, “depression”, “K-10”, “PHQ-9” in various combinations with “rural”, “regional”, “remote”, “Australia”. Snowballing was also used to find relevant literature. Studies were excluded if they only targeted a specific age group, gender, population group, or if a full text could not be accessed. Most studies were cross-sectional in nature and identified age and gender differences or the role of accessibility in the prevalence of mental health disorders. The table shows that under-representation of rural and regional participants is a key limitation of the previous population surveys on mental health in Australia^

**S2. Adjusted odd ratio (aOR) of factors associated with threshold level distress among rural/regional Victorians in Crossroads II study** (2016-18, *n*=741)**.**

| **Demographics** | **Model 1** | **Model 2** | **Model 3A** | **Model 3B** |
| --- | --- | --- | --- | --- |
| **Age group in years** | aOR [95%CI] | aOR [95%CI] | aOR [95%CI] | aOR [95%CI] |
| ≤34 | *Ref* | *Ref* | *Ref* | *Ref* |
| 35-54 | 0.66 [0.26, 1.71] | 0.50 [0.17, 1.42] | 0.50 [0.17, 1.43] | 0.45 [0.16, 1.31] |
| ≥55 | 0.41 [0.15, 1.07] | 0.62 [0.22, 1.76] | 0.70 [0.24, 2.06] | 0.54 [0.18, 1.55] |
| **Sex** |  |  |  |  |
| Male | *Ref* | *Ref* | *Ref* | *Ref* |
| Female | 1.35 [0.76, 2.42] | 1.47 [0.77, 2.80] | 1.39 [0.71, 2.71] | 1.65 [0.85, 3.20] |
| Others |  |  |  |  |
| **Location*** |  |  |  |  |
| Rural | *Ref* | *Ref* | *Ref* | *Ref* |
| Regional | 1.70 [0.95, 3.03] | 1.60 [0.85, 3.00] | 1.69 [0.89, 3.19] | 1.60 [0.85, 3.00] |
| **Employment Status** |  |  |  |  |
| Working full time | *Ref* | *Ref* | *Ref* | *Ref* |
| Working part time | **0.43 [0.20, 0.95]** | 0.48 [0.21, 1.11] | 0.48 [0.21, 1.12] | 0.53 [0.23, 1.22] |
| Unemployed | 1.03 [0.52, 2.03] | 0.95 [0.46, 1.95] | 0.95 [0.45, 1.97] | 0.98 [0.47, 2.05] |
| **Highest Education Attained** |  |  |  |  |
| Completed secondary education or less | *Ref* | *Ref* | *Ref* | *Ref* |
| Completed trade/certificate/diploma | 0.78 [0.40, 1.53] | 0.93 [0.45, 1.95] | 0.90 [0.42, 1.90] | 0.94 [0.45, 1.97] |
| Completed university | 0.54 [0.26, 1.15] | 1.02 [0.45, 2.30] | 1.00 [0.44, 2.28] | 1.15 [0.50, 2.65] |
| **Marital Status** |  |  |  |  |
| Married/De-facto | *Ref* | *Ref* | *Ref* | *Ref* |
| Unmarried | **2.12 [1.21, 3.74]** | 1.38 [0.74, 2.57] | 1.38 [0.71, 2.52] | 1.28 [0.68, 2.40] |
| Ethnicity |  |  |  |  |
| Australian-born | *Ref* | *Ref* | *Ref* | *Ref* |
| non-Australian born | 1.51 [0.75, 3.05] | 1.68 [0.78, 3.62] | 1.74 [0.79, 3.85] | 1.71 [0.79, 3.72] |
| **Indigenous Status** |  |  |  |  |
| Aboriginal and/or Torres Strait Islander |  |  |  |  |
| No | *Ref* | *Ref* | *Ref* | *Ref* |
| Yes | **5.74 [1.19, 27.6]** | 5.31 [0.98, 28.8] | 5.45 [0.96, 30.78] | 4.73 [0.90, 24.9] |
| **Income factors** |  |  |  |  |
| **Earns income** |  |  |  |  |
| Yes | *Ref* | *Ref* | *Ref* | *Ref* |
| No | 1.05 [0.42, 2.65] | 1.59 [0.59, 4.29] | 1.58 [0.57, 4.36] | 1.58 [0.58, 4.35] |
| *Main source of income* |  |  |  |  |
| Wages or salary/others | *Ref* | *Ref* | *Ref* | *Ref* |
| Pension/superannuation | 1.53 [0.74, 3.17] | 1.59 [0.73, 3.45] | 1.44 [0.65, 3.16] | 1.54 [0.71, 3.33] |
| Own business/investment | 0.51 [0.14, 1.85] | 0.71 [0.18, 2.81] | 0.62 [0.15, 2.55] | 0.70 [0.17, 2.83] |
| **Lifestyle factors** |  |  |  |  |
| **BMI,** kg/m2 |  |  |  |  |
| Underweight/Healthy Weight (≤24.9) |  | *Ref* | *Ref* | *Ref* |
| Overweight |  | 1.14 [0.49, 2.66] | 1.18 [0.50, 2.78] | 1.17 [0.50, 2.76] |
| Obese (≥30) |  | **2.74 [1.26, 5.95]** | **2.72 [1.23, 6.03]** | **2.33 [1.05, 5.15]** |
| **Smoking** |  |  |  |  |
| Non-smoker |  | *Ref* | *Ref* | *Ref* |
| Current smoker |  | **3.53 [1.46, 8.53]** | **3.55 [1.47, 8.56]** | **3.25 [1.34, 7.88]** |
| **Alcohol Consumption (yes/no)** |  |  |  |  |
| None |  | *Ref* | *Ref* | *Ref* |
| <4 drinks |  | 0.60 [0.28, 1.27] | 0.61 [0.28, 1.31] | 0.64 [0.30, 1.37] |
| 4+drinks |  | 0.88 [0.31, 2.51] | 0.91 [0.31, 2.62] | 1.03 [0.36, 2.99] |
| **Physical activity** |  |  |  |  |
| None |  | *Ref* | *Ref* | *Ref* |
| Inadequate |  | 0.76 [0.35, 1.67] | 0.72 [0.32, 1.61] | 0.78 [0.35, 1.70] |
| Adequate |  | 0.59 [0.28, 1.21] | 0.59 [0.28, 1.24] | 0.62 [0.30, 1.29] |
| Community Participation |  |  |  |  |
| No |  | *Ref* | *Ref* | *Ref* |
| Yes |  | **0.53 [0.28, 0.99]** | **0.52 [0.28, 0.98]** | 0.54 [0.29, 1.01] |
| Health factors |  |  |  |  |
| **Diabetes status** |  |  |  |  |
| No |  |  | *Ref* |  |
| Yes |  |  | 0.59 [0.18, 1.93] |  |
| Asthma |  |  |  |  |
| No |  |  | *Ref* |  |
| Yes |  |  | 1.66 [0.75, 3.65] |  |
| Emphysema |  |  |  |  |
| No |  |  | *Ref* |  |
| Yes |  |  | 1.86 [0.32, 10.8] |  |
| Stroke |  |  |  |  |
| No |  |  | *Ref* |  |
| Yes |  |  | 1.07 [0.19, 13.6] |  |
| Heart disease |  |  |  |  |
| No |  |  | *Ref* |  |
| Yes |  |  | 1.63 [0.20, 13.6] |  |
| **Self-rated general health*** |  |  |  |  |
| Very good/excellent |  |  |  | *Ref* |
| Poor/good |  |  |  | **2.18 [1.06, 4.47]** |

^CI, confidence intervals of the odd ratios are shown in parenthesis. Psychological distress was derived from K-10 questionnaire, depression was derived from PhQ-9 questionnaire. BMI, body mass index *Regional include Shepperton and Mooroopna towns while rural included Benalla, Cobram, and Seymour towns^*^. Significant variables are bolder.^* ^Empty cells are variables not included in the specific model. Model 1: demographic factors of age group, sex, location, employment status, highest education attained, marital status, ethnicity, aboriginal status, income factors; Model 2: Model 1 plus life style factors of body mass index (BMI), smoking status, alcohol consumption, physical activity; and community participation; Model 3A: Model 2 plus health factors including diabetes, asthma, stroke, heart disease, emphysema while Model 3B: Model 2 plus and self-rated health.^

**S3. Adjusted odd ratio (aOR) of factors associated with threshold-level depression using PHQ-9 among rural/regional Victorians in Crossroads II study** (2016-18, *n*=701)**.**

| **Demographics** | **Model 1** | **Model 2** | **Model 3A** | **Model 3B** |
| --- | --- | --- | --- | --- |
| **Age group in years** | aOR [95%CI] | aOR [95%CI] | aOR [95%CI] | aOR [95%CI] |
| ≤34 | *Ref* | *Ref* | *Ref* | *Ref* |
| 35-54 | 1.16 [0.43, 3.17] | 1.02 [0.35, 3.02] | 1.02 [0.34, 3.04] | 0.85 [0.29, 2.59] |
| ≥55 | 0.43 [0.15, 1.22] | 0.61 [0.19, 1.90] | 0.63 [0.19, 2.04] | 0.45 [0.14, 1.46] |
| **Sex** |  |  |  |  |
| Male | *Ref* | *Ref* | *Ref* | *Ref* |
| Female | 0.93 [0.51, 1.71] | 0.94 [0.48, 1.84] | 0.94 [0.47, 1.89] | 1.13 [0.57, 2.24] |
| Others |  |  |  |  |
| **Location*** |  |  |  |  |
| Rural | *Ref* | *Ref* | *Ref* | *Ref* |
| Regional | **2.03 [1.09, 3.78]** | 1.80 [0.92, 3.52] | 1.82 [0.92, 3.60] | 1.74 [0.88, 3.45] |
| **Employment Status** |  |  |  |  |
| Working full time | *Ref* | *Ref* | *Ref* | *Ref* |
| Working part time | 0.78 [0.36, 1.70] | 1.00 [0.43, 2.33] | 1.01 [0.43, 2.37] | 1.17 [0.49, 2.78] |
| Unemployed | 1.41 [0.67, 2.97] | 1.32 [0.60, 2.92] | 1.27 [0.57, 2.85] | 1.53 [0.67, 3.45] |
| **Highest Education Attained** |  |  |  |  |
| Completed secondary education or less | *Ref* | *Ref* | *Ref* | *Ref* |
| Completed trade/certificate/diploma | 1.43 [0.72, 2.86] | 1.67 [0.79, 3.53] | 1.70 [0.79, 3.63] | 1.65 [0.77, 3.54] |
| Completed university | 0.72 [0.31, 1.65] | 1.49 [0.60, 3.73] | 1.44 [0.57, 3.64] | 1.87 [0.73, 4.81] |
| **Marital Status** |  |  |  |  |
| Married/De-facto | *Ref* | *Ref* | *Ref* | *Ref* |
| Unmarried | **3.54 [1.92, 6.53]** | **2.81 [1.45, 5.44]** | **2.75 [1.41, 5.37]** | **2.56 [1.30, 5.03]** |
| Ethnicity |  |  |  |  |
| Australian-born | Ref | Ref | Ref | Ref |
| non-Australian born | 0.67 [0.28, 1.59] | 0.63 [0.24, 1.61] | 0.61 [0.23, 1.61] | 0.63 [0.24, 1.65] |
| **Indigenous Status** |  |  |  |  |
| Aboriginal and/or Torres Strait Islander |  |  |  |  |
| No | *Ref* | *Ref* | *Ref* | *Ref* |
| Yes | 2.38 [0.44, 12.8] | 2.36 [0.38, 14.55] | 2.32 [0.37, 14.63] | 2.26 [0.39, 13.20] |
| **Income factors** |  |  |  |  |
| **Earns income** |  |  |  |  |
| Yes | *Ref* | *Ref* | *Ref* | *Ref* |
| No | 0.64 [0.23, 1.77] | 0.76 [0.25, 2.33] | 0.69 [0.22, 2.17] | 0.72 [0.23, 2.27] |
| *Main source of income* |  |  |  |  |
| Wages or salary/others | *Ref* | *Ref* | *Ref* | *Ref* |
| Pension/superannuation | 1.49 [0.69, 3.20] | 1.51 [0.67, 3.41] | 1.38 [0.60, 3.17] | 1.41 [0.62, 3.21] |
| Own business/investment | 0.56 [0.15, 2.08] | 0.78 [0.19, 3.19] | 0.68 [0.16, 2.89] | 0.75 [0.17, 3.21] |
| **Lifestyle factors** |  |  |  |  |
| **BMI,** kg/m2 |  |  |  |  |
| Underweight/Healthy Weight (≤24.9) |  | *Ref* | *Ref* | *Ref* |
| Overweight |  | 2.28 [0.85, 6.10] | 2.44 [0.89, 6.68] | 2.40 [0.88, 6.56] |
| Obese (≥30) |  | **3.67 [1.44, 9.37]** | **3.79 [1.42, 10.1]** | **2.96 [1.14, 7.70]** |
| **Smoking** |  |  |  |  |
| Non-smoker |  | *Ref* | *Ref* | *Ref* |
| Current smoker |  | 1.53 [0.60, 3.93] | 1.55 [0.60, 3.99] | 1.36 [0.52, 3.48] |
| **Alcohol Consumption (yes/no)** |  |  |  |  |
| None |  | *Ref* | *Ref* | *Ref* |
| <4 drinks |  | 0.57 [0.25, 1.29] | 0.55 [0.24, 1.28] | 0.62 [0.27, 1.44] |
| 4+drinks |  | 0.83 [0.28, 2.48] | 0.81 [0.27, 2.44] | 1.04 [0.34, 3.14] |
| **Physical activity** |  |  |  |  |
| None |  | *Ref* | *Ref* | *Ref* |
| Inadequate |  | 0.78 [0.35, 1.73] | 0.78 [0.35, 1.78] | 0.81 [0.36, 1.81] |
| Adequate |  | **0.39 [0.18, 0.86]** | **0.42 [0.19, 0.92]** | **0.42 [0.19, 0.93]** |
| Community Participation |  |  |  |  |
| No |  | *Ref* | *Ref* | *Ref* |
| Yes |  | **0.49 [0.25, 0.96]** | 0.51 [0.26, 1.03] | 0.52 [0.26, 1.02] |
| **Health factors** |  |  |  |  |
| **Diabetes status** |  |  |  |  |
| No |  |  | *Ref* |  |
| Yes |  |  | 0.82 [0.27, 2.47] |  |
| Asthma |  |  |  |  |
| No |  |  | *Ref* |  |
| Yes |  |  | 1.32 [0.56, 3.14] |  |
| Emphysema |  |  |  |  |
| No |  |  | *Ref* |  |
| Yes |  |  | 4.15 [0.61, 28.17] | |
| Stroke |  |  |  |  |
| No |  |  | *Ref* |  |
| Yes |  |  | 0.39 [0.04, 4.03] |  |
| Heart disease |  |  |  |  |
| No |  |  | *Ref* |  |
| Yes |  |  | 2.02 [0.22, 18.28] | |
| **Self-rated general health*** |  |  |  |  |
| Very good/excellent |  |  |  | *Ref* |
| Poor/good |  |  |  | **3.58 [1.54, 8.34]** |

^CI, confidence intervals of the odd ratios are shown in parenthesis. Psychological distress was derived from K-10 questionnaire, depression was derived from PhQ-9 questionnaire. BMI, body mass index *Regional include Shepperton and Mooroopna towns while rural included Benalla, Cobram, and Seymour towns^*^. Significant variables are bolder.^* ^Empty cells are variables not included in the specific model. Model 1: demographic factors of age group, sex, location, employment status, highest education attained, marital status, ethnicity, aboriginal status, income factors; Model 2: Model 1 plus life style factors of body mass index (BMI), smoking status, alcohol consumption, physical activity; and community participation; Model 3A: Model 2 plus health factors including diabetes, asthma, stroke, heart disease, emphysema while Model 3B: Model 2 plus and self-rated health.^
